# Iron Deficiency Induces a Partial Inhibition of the Photosynthetic Electron Transport and a High Sensitivity to Light in the Diatom *Phaeodactylum tricornutum*

**DOI:** 10.3389/fpls.2016.01050

**Published:** 2016-08-03

**Authors:** Mercedes Roncel, Antonio A. González-Rodríguez, Belén Naranjo, Pilar Bernal-Bayard, Anna M. Lindahl, Manuel Hervás, José A. Navarro, José M. Ortega

**Affiliations:** Instituto de Bioquímica Vegetal y Fotosíntesis, Universidad de Sevilla and Consejo Superior de Investigaciones CientíficasSeville, Spain

**Keywords:** iron deficiency, light sensitivity, PAM fluorescence, *Phaeodactylum tricornutum*, photosystem II, thermoluminescence

## Abstract

Iron limitation is the major factor controlling phytoplankton growth in vast regions of the contemporary oceans. In this study, a combination of thermoluminescence (TL), chlorophyll fluorescence, and P700 absorbance measurements have been used to elucidate the effects of iron deficiency in the photosynthetic electron transport of the marine diatom *P. tricornutum*. TL was used to determine the effects of iron deficiency on photosystem II (PSII) activity. Excitation of iron-replete *P. tricornutum* cells with single turn-over flashes induced the appearance of TL glow curves with two components with different peaks of temperature and contributions to the total signal intensity: the B band (23°C, 63%), and the AG band (40°C, 37%). Iron limitation did not significantly alter these bands, but induced a decrease of the total TL signal. Far red excitation did not increase the amount of the AG band in iron-limited cells, as observed for iron-replete cells. The effect of iron deficiency on the photosystem I (PSI) activity was also examined by measuring the changes in P700 redox state during illumination. The electron donation to PSI was substantially reduced in iron-deficient cells. This could be related with the important decline on cytochrome *c*_6_ content observed in these cells. Iron deficiency also induced a marked increase in light sensitivity in *P. tricornutum* cells. A drastic increase in the level of peroxidation of chloroplast lipids was detected in iron-deficient cells even when grown under standard conditions at low light intensity. Illumination with a light intensity of 300 μE m^-2^ s^-1^ during different time periods caused a dramatic disappearance in TL signal in cells grown under low iron concentration, this treatment not affecting to the signal in iron-replete cells. The results of this work suggest that iron deficiency induces partial blocking of the electron transfer between PSII and PSI, due to a lower concentration of the electron donor cytochrome *c*_6_. This decreased electron transfer may induce the over-reduction of the plastoquinone pool and consequently the appearance of acceptor side photoinhibition in PSII even at low light intensities. The functionality of chlororespiratory electron transfer pathway under iron restricted conditions is also discussed.

## Introduction

Studies on the primary productivity of phytoplankton have revealed that iron (Fe) limitation is the major factor controlling phytoplankton growth in vast regions of the contemporary oceans (“iron hypothesis”), most notably in the high nutrient low chlorophyll regions (HNLC) (Martin and Fitzwater, 1988; Boyd et al., 2000; de Baar and Boyd, 2000; Dalton, 2002; Gervais et al., 2002; Moore et al., 2007). Fe is an essential micronutrient for phytoplankton because it is a cofactor of proteins directly involved in photosynthesis, respiration, nitrate, nitrite and sulfate reduction, N_2_ fixation, chlorophyll synthesis, and a number of other biosynthetic or degradative reactions (Geider et al., 1993; Geider and La Roche, 1994; Morel and Price, 2003). Fe plays a crucial role as component of different photosynthetic electron carriers as cytochrome (Cyt) *b*_6_*f* and Cyt *c*_6_ and iron–sulfur complexes and as an integral part of both photosystem I (PSI) and photosystem II (PSII; Greene et al., 1991, 1992; McKay et al., 1997; Erdner and Anderson, 1999).

Iron deficiency causes important alterations in thylakoid membrane structure and the basic processes involved in photochemical energy conversion. A general decrease in transcripts associated with photosynthesis has been shown under this stress condition (Allen et al., 2008). Chlorosis is one of the most important symptoms of Fe deficiency (Greene et al., 1991; Davey and Geider, 2001; Moseley et al., 2002; Allen et al., 2008). It is attributed to the inhibition of the chlorophyll (Chl) biosynthetic pathway, which requires the function of iron-containing enzymes (van Leeuwe and Stefels, 1998, 2007). Disconnection between light-harvesting centers as a consequence of ultrastructure changes of the thylakoid membranes under Fe-limited conditions (Meisch et al., 1980; Terry and Abadía, 1986; Hilt et al., 1987) has been described as responsible for a decline of PSII efficiency, electron transport and carbon fixation (Raven, 1990; Behrenfeld and Milligan, 2012; Petrou et al., 2014). Reduced photosynthetic efficiency due to loss of the D1 protein (Greene et al., 1992), reduced concentration of Cyt *b*_6_*f* and Cyt *c*_6_ and decreasing Cyt *f*:Chl *a*, P700:Chl *a*, and PSI:PSII ratios (Greene et al., 1991; Allen et al., 2008) have been observed in Fe-limited continuous cultures of the diatom *P. tricornutum*.

Diatoms (Bacillariophyceae) are the most important eukaryotic group of phytoplankton (Guiry, 2012) with a key role in influencing the global climate since they are responsible for up to 20% of the global primary productivity and 40% of the carbon sequestration in the oceans (Treguer et al., 1995; Field et al., 1998; Bowler et al., 2010; Falkowski and Raven, 2013). Fe fertilization experiments in HNLC regions have shown the appearance of blooms dominated by diatoms, suggesting that these algae have adaptations that allow survival in Fe limited waters and a subsequent rapid multiplication when Fe becomes available (Morrissey and Bowler, 2012). Different strategies have been developed by diatoms to minimize their Fe requirements: a decrease of the cellular pigment concentrations at the cost of light capture efficiency (Petrou et al., 2011), biochemical alteration of the photosynthetic Fe demand through decreased expression of the Fe-rich PSI and Cyt *b*_6_*f* components (Strzepek and Harrison, 2004; Allen et al., 2008) and/or substitute Fe-containing enzymes as ferredoxin by proteins with Fe-free equivalents, as flavodoxin (La Roche et al., 1996; Marchetti et al., 2009). Diatoms have also developed specific regulatory mechanisms to dissipate energy excess under environmental stress conditions (Grouneva et al., 2009; Goss and Jakob, 2010; Lavaud and Goss, 2014).

In this study, we have investigated the photosynthetic response of the pennate marine diatom *P. tricornutum* to Fe deficiency, using thermoluminescence (TL), Chl fluorescence, and P700 redox state measurements. These techniques are very simple, precise and non-destructive, and provide valuable *in vivo* measurements of the effects of environmental perturbations on PSII and PSI activity (Ducruet, 2003; Allen et al., 2008; Kalaji et al., 2014b). Chl *a* fluorescence technique has been extensively used for studying the effects of different environmental stresses on photosynthesis (Maxwell and Johnson, 2000; Kalaji et al., 2011, 2014b; Goltsev et al., 2012).

*Phaeodactylum tricornutum* is highly tolerant to Fe limitation and can grow in steady-state laboratory cultures at Fe levels 50 times lower than those tolerated by others diatoms (Kustka et al., 2007). Studies on this diatom have shown that it is also unusually resistant to damage by exposure to high light intensities (Olaizola et al., 1994). Moreover, this organism has a xanthophyll-dependent non-photochemical quenching (NPQ) that is induced more rapidly, and can compete with excitation transfer to the PSII reaction center (RC), much more efficiently than does the analogous process in higher plants (Lavaud et al., 2002a). In addition, *P. tricornutum* may be able to short-circuit its PSII RC by a cyclic electron transfer path when the charge separation cannot be stabilized by normal secondary electron transport (Lavaud et al., 2002b).

Thermoluminescence provides an *in vivo* measure of the response of PSII activity to environmental stresses (Rahoutei et al., 1990; Briantais et al., 1992; Misra et al., 1997; Walters and Johnson, 1997; Roman and Ducruet, 2000). Photosynthetic luminescence is a process that originates from PSII by recombination of charge pairs separated by a prior irradiation. Luminescence decay phases can be better resolved by TL emission technique, which consists in recording luminescence emission during the warming of a sample after an irradiation given at a relatively low temperature (for a review, see Vass and Inoue, 1992; Inoue, 1996; Ducruet, 2003). Therefore, the properties of the two principal TL emission bands, B band and AG band (Ducruet, 2003), may be used to obtain information on the effect of Fe deficiency on the photochemical activity of PSII. The B band is the result of the recombination of S_2_/S_3_Q_B_^-^ pairs, Q_B_ being the secondary quinone acceptor, and S_2_/S_3_ being the states of the oxygen-evolving complex (OEC) storing two or three positive charges (Rutherford et al., 1984; Vass and Inoue, 1992). The AG band, although originating from PSII, is governed by the electron back-transfer from the stroma to Q_B_, which requires (i) a sufficient potential gap between the acceptors side of PSI (NADPH/NADP) and PSII (PQH_2_/PQ); (ii) an activated chlororespiratory pathway involving both non-photochemical reduction and oxidation of plastoquinones (PQs) (Bennoun, 1982; Sundblad et al., 1988; Rumeau et al., 2007).

Chlororespiration has been defined as a respiratory electron transport chain in interaction with the photosynthetic electron transfer in thylakoid membranes of chloroplasts. It involves mostly a NAD(P)H-PQ oxidoreductase activity (Ndh activity), the thylakoid PQ pool and a terminal oxidase named PTOX (Peltier and Cournac, 2002). A chlororespiratory reduction of the PQ pool has been found in diatoms and algae (Wilhelm and Duval, 1990; Dijkman and Kroon, 2002) leading to the build-up of a proton gradient without the participation of PSII electron transport (Jakob et al., 1999, 2001). Several studies have also proposed that chlororespiratory components may be involved in protective or adaptive mechanisms of photosynthetic organisms to environmental stress conditions (Rumeau et al., 2007).

In this work, a combination of TL, Chl fluorescence and P700 (PSI primary donor) absorbance measurements have been used to elucidate the effects of Fe deficiency in the photosynthetic electron transport activity of *P. tricornutum*. The results obtained in this work suggest that Fe deficiency induces the partial blocking of electron transfer from PSII to PSI, and consequently, leads to a more reduced state of the PQ pool. This blocking is likely to be due to an important reduction of the amount of Cyt *c*_6_. Fe deficiency induced also a significant increase of the light sensitivity of PSII. The possible activation under low Fe concentration of alternative secondary electron transfer pathways, as chlororespiration, is discussed.

## Materials and Methods

### Cell Culture Conditions

The experiments described in this work were carried out using cells from the coastal diatom *P. tricornutum* CCAP 1055/1. Cells were grown in Artificial Seawater (ASW) medium (McLachlan, 1964; Goldman and McCarthy, 1978) in a rotatory shaker (50 rpm) at 20°C. The cultures were illuminated by fluorescent white lamps at an intensity of 20 μE m^-2^ s^-1^ under a light/dark cycle of 16/8 h. For the experiments of the effects of Fe deficiency, cells from ASW cultures were pelleted at 5000 × *g* for 5 min and grown in standard ASW medium (Fe-replete culture; 12 μM Fe) and ASW medium with only 0.12 μM Fe (Fe-deficient culture). Most of the experiments were carried out using cells from 21 days cultures, with an optical density at 750 nm of 0.93–1.05 and 0.58–0.65 for Fe-replete and Fe-deficient cultures, respectively.

### Chlorophyll and Cytochrome *c*_6_ Content

Chlorophyll *a* and *c* content in *P. tricornutum* cells was determined in acetone solution by differential absorbance measurements. Cells (1 mL) were pelleted at 5000 × *g* for 5 min and the wet pellets were weighed. Precipitated cells were then suspended in acetone 90% (1 mL) and disrupted mechanically in the presence of 0.5 mm diameter glass beads (1 mL; BioSpec Products) by 1 min of agitation (3450 oscillations/min) in a Mini-BeadBeater-16 cell disruptor (BioSpec Products). Cell extracts were spun at 16000 × *g* for 5 min and the supernatants, in which the pigments were extracted, were used to determinate Chl concentrations. Chl *a* and Chl *c* concentrations were measured spectrophotometrically (JASCO V-650 UV-Vis/NIR spectrophotometer, Japan) using the following equations as described by Jeffrey and Humphrey (1975):

$$Chl\,\;a\,\;(mg/L)=11.47\times (A_{664}-A_{750})-0.40\times \left(A_{630}-A_{759}\right)$$

$$Chl\,\;c\,\;(mg/L)=24.34\times (A_{630}-A_{750})-0.40\times \left(A_{664}-A_{759}\right)$$

Cytochrome *c*_6_ content in *P. tricornutum* cells was determined in soluble cell fractions by differential absorbance measurements using a JASCO V-650 spectrophotometer. *P. tricornutum* cells from 100 mL cultures were precipitated by centrifugation at 5000 × *g* for 5 min and wet pellets were weighed. Cells were then suspended to 1 mL in culture media and disrupted by six cycles of freezing in liquid nitrogen and thawing at 40°C in a thermoblock. Soluble fractions were obtained by centrifugation at 16000 × *g* for 15 min to precipitate membranes and cell debris. This method extracted up to 90% of Cyt *c*_6_, as determined by further protein extraction by sonication of the membrane fractions. The total content of Cyt *c*_6_ was estimated from the absorbance difference at 552 nm between the fully reduced (sodium ascorbate, 2 mM) and fully oxidized (potassium ferricyanide, 1 mM) state, using a differential extinction coefficient (reduced minus oxidized) of 15 mM^-1^ cm^-1^ at 552 nm. The amount of Cyt *c*_6_ was related to grams of cell wet weight.

### Immunodetection of Cytochrome *c*_6_

Polyclonal antibodies raised against *P. tricornutum* Cyt *c*_6_ were generated using standard procedures at the Animal Experimentation Facility (University of Seville, Spain) by subcutaneous injection of 1 mg of purified Cyt *c*_6_ protein into a white New Zealand rabbit (Bernal-Bayard et al., 2013). Polyclonal antibodies against the Rubisco large subunit (Agrisera, Sweden) were also used as loading control. About 8.8 × 10^8^ *P. tricornutum* cells from 150 mL cultures, grown under Fe-replete or Fe-deficient conditions, were harvested by centrifugation (5000 × *g* for 5 min). Cells were suspended in lysis buffer, containing 50 mM Tris-HCl (pH 6.8) and 2% SDS, and incubated 30 min at 4°C. The soluble fraction was obtained by centrifugation at 12000 × *g* for 30 min at 4°C. Then, 20 μg of total protein were resolved on 15% (w/v) polyacrylamide gel electrophoresis and transferred to a nitrocellulose membrane (Amersham Protran Premium 0.45 μm NC, GE Healthcare Life Sciences). The membrane was incubated overnight with rabbit anti-Cyt *c*_6_ primary antibody (dilution 1:1000) followed by 1 h incubation with Goat Anti-Rabbit IgG (H+L)-HRP Conjugate (Bio-Rad; dilution 1:10000), and visualized with the Immobilon Western Chemiluminescent HRP Substrate (Millipore).

### Oxygen Evolution

Oxygen evolution and consumption by *P. tricornutum* cell suspensions were measured by polarography using a Clark-type oxygen electrode (Hansatech) at 25°C with saturating and continuous white light (2000 μE m^-2^ s^-1^). Typically, *P. tricornutum* cell suspensions (equivalents to 100 μg Chl) were dark-incubated for 2 min at 25°C and illuminated at the end of this period to measure oxygen evolution or consumption.

### Thermoluminescence

Thermoluminescence glow curves of *P. tricornutum* cell suspensions were obtained using an home-built apparatus designed by Dr. Jean-Marc Ducruet (France) for luminescence detection from 1 to 80°C (standard thermoluminescence, STL) and from 10 to 160°C (high temperature thermoluminescence, HTL). A detailed description of the system can be obtained elsewhere (Ducruet, 2003; Zurita et al., 2005; Guerrero et al., 2014; Repetto et al., 2015). Briefly, temperature regulation, signal recording and flash sequences were driven by a computer through a National Instrument DAQ-Pad 1200 interface, using a specially developed acquisition program (Ducruet, 2003). The sample cuvette consisted in a horizontal chamber (2 cm diameter) with a copper film on the bottom. A double-stage Marlow thermoelectric Peltier plate (model DT 1089-14; Marlow Industries, USA), powered by a variable (0 to 5 A) computer-driven power supply, was mounted below the chamber for temperature regulation. The Peltier element was cooled by a temperature-controlled bath. Luminescence emission was detected by a H5701-50 Hamamatsu photomultiplier module. Illumination was performed through a light guide parallel to the photomultiplier, both of them being attached to the same stand sliding horizontally from the illumination to the measuring position. Single turn-over flashes were provided by a xenon white light (Walz XST-103). Data acquisition, signal analysis and graphical simulation were performed as previously described (Ducruet and Miranda, 1992; Zurita et al., 2005; Ducruet et al., 2011).

Typically, for STL measurements *P. tricornutum* cell suspensions (equivalents to 15 μg Chl) were dark-incubated for 2 min at 20°C, then cooled to 1°C for 1 min and illuminated at the end of this period with different numbers of saturating single turn-over flashes (separated by 1 s). Luminescence emission was then recorded while warming samples from 1 to 80°C at a heating rate of 0.5°C per second. In some experiments, before recording the luminescence emissions, white or far red (FR) light illuminations were applied through a optic fiber to cell suspensions by using a tungsten lamp non-filtered (300 μE m^-2^s^-1^ light intensity) or filtered through a 695 nm cut-off filter (4 μE m^-2^s^-1^ light intensity), respectively.

For HTL measurements *P. tricornutum* cell suspensions (equivalents to 7.5 μg Chl) were adsorbed by filtration on a piece of filter paper (0.45 μm, Whatman) that was pressed against the copper film, dark-incubated for 10 min at 20°C and cooled to 10°C for 1 min. Luminescence emission was then recorded while warming samples from 10 to 160°C at a heating rate of 0.1°C per second. N_2_ gas was flushed on the sample during HTL experiments in order to desiccate samples and prevents any oxidation induced by high temperatures.

Standard thermoluminescence and HTL experiments were repeated five times. The experiments shown in **Figures 2–5** are representative examples.

### Chlorophyll *a* Fluorescence and Photosystem I P700 Redox State

Room temperature Chl *a* fluorescence was measured using a pulse-amplitude modulation fluorometer (DUAL-PAM-100, Walz, Effeltrich, Germany). The maximum quantum yield of PSII was assayed after incubation of the cell suspension in the dark for 30 min by calculating the ratio of the variable fluorescence, *F*_v_, to maximal fluorescence, *F*_m_, (*F*_v_/*F*_m_). Relative linear electron transport rates (rETR) were measured in pre-illuminated cell suspensions applying stepwise increasing red (635 nm) actinic light intensities up to 2000 μE m^-2^ s^-1^. Effective PSII quantum yield for each actinic light intensity was determined using saturating pulses of red light at 10000 μE m^-2^ s^-1^ intensity and 0.6 s duration. The effective PSII quantum yield Y(II) and relative linear electron transport rates were calculated by the DUAL-PAM-100 software according to the equations by Kramer et al. (2004).

The redox state of PSI P700 was monitored by following changes in absorbance at 830 nm versus 875 nm using the DUAL-PAM-100 apparatus. Cells were incubated in darkness for 30 min prior to measurements. To probe the maximum extent of P700 oxidation, cell suspensions were illuminated with FR (730 nm) light for 10 s, thereafter a saturating pulse of red (635 nm) light at 10000 μE m^-2^ s^-1^ intensity and 0.6 s duration was applied. Following the determination of maximal oxidation of P700, the actinic red (635 nm) light at an intensity of 126 μE m^-2^ s^-1^ was switched on and saturating pulses were applied every 20 s. After 5 min, the actinic light was switched off. The respective quantum yields of PSI photochemistry, Y(I), donor side limitations, Y(ND), and acceptor side limitations, Y(NA), were calculated by the DUAL-PAM-100 software.

## Results

The effects of Fe deficiency on various physiological and biochemical parameters of *P. tricornutum* cells have been investigated. **Table 1** shows the results obtained for these parameters in cells harvested after 21 days of growing in both Fe-replete and Fe-deficient conditions under our experimental conditions. After 1 week of Fe limitation, growth gradually slowed down (see Supplementary Figure **S1**). A significant decrease of the growth rates in Fe-deficient *P. tricornutum* cells was observed after 21 days of cultures in comparison with cells cultured in Fe-replete conditions (**Table 1**). Fe-deficient *P. tricornutum* cells showed lower concentration of Chl *a* (56%). Fe deficiency also induced a significant decrease of about 60 and 80% in the oxygen evolving and respiration activities of the cells, respectively. Overall, the effects observed under Fe limitation are consistent with those described previously (Kudo et al., 2000; Allen et al., 2008).

**Table 1:** Physiological and biochemical parameters of *Phaeodactylum tricornutum* cells from Fe-replete and Fe-deficient cultures.

Parameter	Fe-replete	Fe-deficient
Growth rate (μ, days^-1^)	0.115 ± 0.014	0.081 ± 0.012
Chl *a* (mg Chl. g^-1^ wwt biomass)	5.5 ± 0.9	3.1 ± 0.7
Chl *c* (mg Chl. g^-1^ wwt biomass)	1.2 ± 0.5	1.3 ± 0.3
O_2_ evolution (μmol O_2_. mg^-1^ Chl. h^-1^)	115 ± 12	51 ± 7
O_2_ consumption (μmol O_2_. mg^-1^ Chl. h^-1^)	59 ± 4	11 ± 4
*F*_v_/*F*_m_	0.615 ± 0.018	0.404 ± 0.023


*Specific growth rates were calculated as μ = (ln OD_2_ – ln OD_1_)/Δt, where OD_1_ and OD_2_ are optical density at 750 nm of cells after 7 and 21 days of culture, respectively, and Δt the corresponding time interval. Chlorophyll concentration is expressed as mg of Chl per g of wet weight biomass (wwt). Data represent the mean ± SD of three replicate determinations from separate cultures. Measurements were done using cells harvested after 21 days of culture.*

**FIGURE 1:**
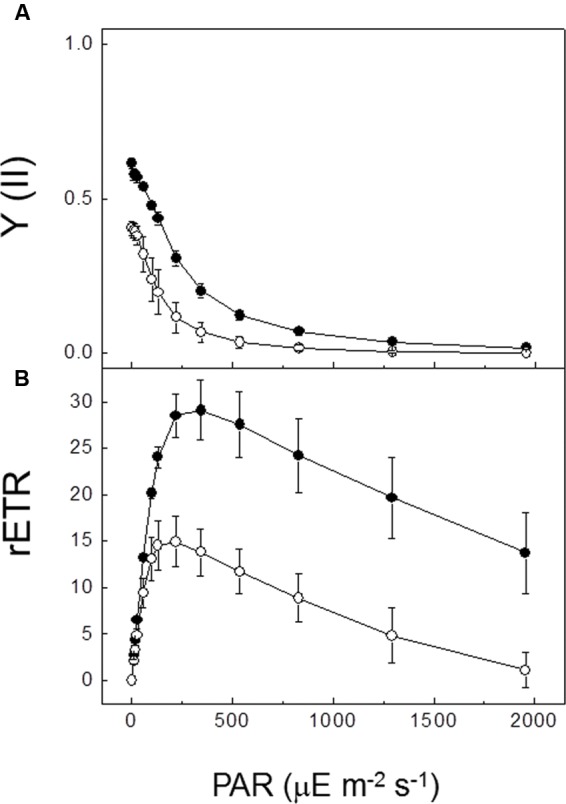
**Effect of iron deficiency on *Chl a* fluorescence parameters of *P. tricornutum*.** **(A)** Quantum yield of PSII photochemistry and **(B)** relative linear electron transport rate in Fe-replete (*filled circles*) and Fe-deficient (*open circles*) cultures of *P. tricornutum* as a function of irradiance derived from steady-state light curves. Chlorophyll fluorescence was measured with a pulse-amplitude modulation fluorometer using Fe-replete and Fe-deficient *P. tricornutum* cultures in exponential phase of grown (21 days). Quantum yield of PSII photochemistry, Y(II), and relative linear electron transport rates, rETR, were determined during stepwise increasing photosynthetically active radiation (PAR) from 0 to 2000 μE m^-2^ s^-1^ light intensity. The curves shown in this figure represent the mean ± SD of three independent experiments.

The effects of Fe deficiency on PSII photochemistry of *P. tricornutum* cells were investigated using Chl *a* fluorescence and TL techniques. Measurements of Chl *a* fluorescence showed clear differences in the photosynthetic activity of PSII between Fe-replete and Fe-deficient cells (**Table 1** and **Figure 1**). The maximum quantum yield of PSII, measured as *F*_v_/*F*_m_, was significantly decreased in Fe-deficient cells. Thus, whereas Fe-replete cultures showed an *F*_v_/*F*_m_ value of 0.615, the value of the Fe-deficient cultures decreased to 0.404 (**Table 1**). Steady-state light curves showed that the effective quantum yield of PSII, Y(II), was lower in Fe-deficient cultures at all irradiance levels tested as compared with Fe-replete cultures (**Figure 1A**). At the maximum irradiance (1957 μE m^-2^ s^-1^), values dropped well below 0.1 under both Fe-culture conditions. The relative electron transport rates (rETR) were significantly greater in the Fe-replete cultures than those measured in the Fe-deficient cultures in the complete range of tested irradiances (**Figure 1B**). The maximal rETR was 50% lower in the Fe-deficient cultures. Taddei et al. (2016) have recently reported the severe decrease of both *F*_v_/*F*_m_ (70%) and rETR (64%) parameters induced by Fe limitation in *P. tricornutum* cells. In Fe-deficient cells rETR was almost completely inhibited at light intensities above 2000 μE m^-2^ s^-1^ (**Figure 1B**). However, in Fe-replete cultures the rETR value measured at this light intensity remained at about 50% of the maximum value. rETR started to decrease above a 200 μE m^-2^ s^-1^ irradiance value in Fe-deficient cells; however, in Fe-replete cells inhibition of electron transfer was observed above irradiance values of 300 μE m^-2^ s^-1^ (**Figure 1B**). Thus, a substantially higher sensitivity to light was observed in *P. tricornutum* cells grown under low Fe concentration.

**FIGURE 2:**
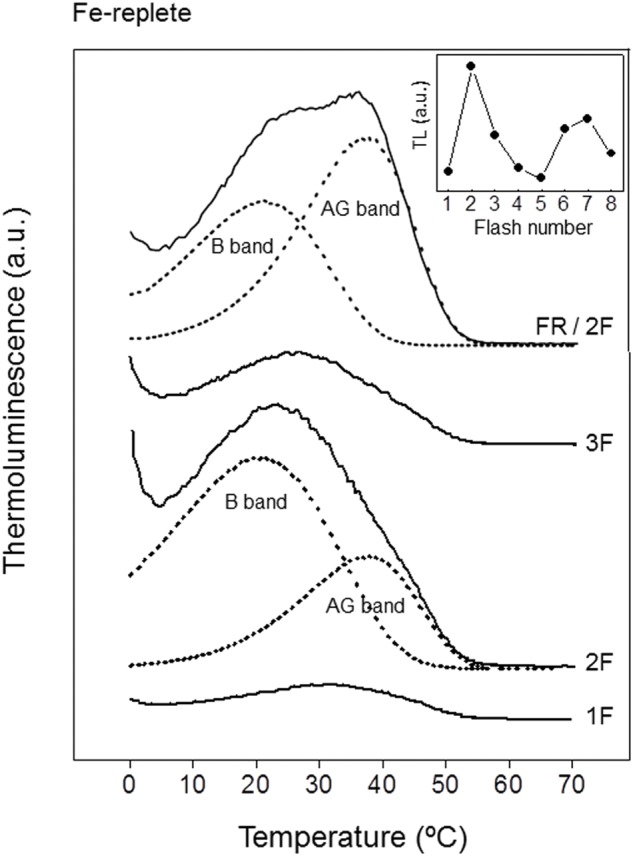
**Thermoluminescence glow curves of *P. tricornutum* cells from Fe-replete cultures.** Typically, cell suspensions (equivalents to 15 μg Chl) from 21 days Fe-replete cultures were incubated in the darkness for 2 min at 20°C, then cooled to 1°C for 1 min and illuminated at the end of this period with different numbers of flashes of white light (1F, 2F, 3F) separated by 1 s. Luminescence emission was then recorded while warming samples from 1 to 80°C at a heating rate of 0.5°C s^-1^. In FR pre-illuminated experiments (FR/2F), before recording the luminescence emissions, FR light illuminations were applied through a optic fiber to cell suspensions using a tungsten lamp filtered through a 695 nm cut-off filter (4 μE m^-2^s^-1^ light intensity). The dashed lines represent the simulation components (B and AG bands) corresponding to the best fit obtained from the deconvolution software used (see Materials and Methods section). *Inset*: Oscillation of the intensity of B band as function of flash number. Intensities were obtained from the component analysis of the curves of TL. For further technical details see “Materials and Methods” section.

Before the analysis of the effects of Fe deficiency on TL emissions of *P. tricornutum* cells, we have characterized some of the most relevant TL bands detected *in vivo* using healthy cells from cultures of this diatom. Not much is known about the characteristics of TL bands in diatoms because only a few TL studies have been carried out previously (Vavilin et al., 2002; Eisenstadt et al., 2008; Materna et al., 2009). Excitation of dark-adapted *P. tricornutum* cells at 1°C with a series of saturating single turn-over flashes induced the appearance of very complex TL glow curves, with differences in the temperature of the maximum (*t*_max_) and signal intensity. TL curves induced by 1, 2, and 3 flashes are shown in **Figure 2** as examples. The light emission curve obtained after illumination with two flashes was the largest of the series and showed a *t*_max_ at about 24°C and a small shoulder around 39°C (**Figure 2**, 2F). These TL signals could be well simulated by two decomposition components, with different *t*_max_ and contributions to the total signal intensity. The decomposition analysis of the emission curve induced by two flashes is shown in **Figure 2** (2F, dotted lines) as an example. This first component can be assigned to the well-known TL B band originating from the recombination reactions of S_3_Q_B_^-^ and S_2_Q_B_^-^ charge pairs in PSII. A *t*_max_ value of 23°C and a signal contribution of 63% were obtained for this band (**Table 2**). We tentatively assigned the second TL component appearing at higher temperatures to the AG band, usually induced by FR illumination in intact photosynthetic materials (Miranda and Ducruet, 1995). A *t*_max_ value of 40°C and a signal contribution of 37% were obtained for this AG band (**Table 2**). A similar AG band has been observed in leaves of pea, *Arabidopsis* and tobacco and in cells of the green alga *Chlamydomonas reinhardtii* excited by white light (Miranda and Ducruet, 1995; Ducruet et al., 2005, 2011; Ducruet and Vass, 2009). This TL emission seems to reflect a back-flow of electrons from unknown reductants present in the stroma to the quinone acceptors of PSII, allowing their recombination with S_2_ and S_3_ states (Sundblad et al., 1988; Miranda and Ducruet, 1995). Whereas recombination of S_2_Q_B_^-^ and S_3_Q_B_^-^ centers produces a B band, the S_2_Q_B_ and S_3_Q_B_ centers should not lead to luminescence emission, unless an electron is progressively fed back to Q_B_, resulting in AG emission. AG band also appears after illumination with continuous white light or flashes in some metabolic conditions: when the use of photosynthetic energy is slowed down due to a lack of CO_2_ (Mellvig and Tillberg, 1986), in young pea leaves (Miranda and Ducruet, 1995) and when CAM metabolism is activated in a CAM-inducible species (Krieger et al., 1998). AG emission has been associated to the activation of cyclic/chlororespiratory electron flows in leaves by stress conditions (Ducruet, 2003).

**Table 2:** Thermoluminescence band emissions of *Phaeodactylum tricornutum* cells from Fe-replete and Fe-deficient cultures.

Conditions		B band	AG band
2F	Fe-replete	23°C (63%)	40°C (37%)
	Fe-deficient	22°C (71%)	41°C (29%)
FR/2F	Fe-replete	21°C (43%)	40°C (57%)
	Fe-deficient	20°C (66%)	39°C (34%)


*Values are the temperature of the intensity maximum of the thermoluminescence components obtained from the deconvolution software used (see Materials and Methods section) and the percentage of total intensity of such components. Values are means of 3–4 replicate determinations from separate cultures.*

Analysis of TL yields in dark-adapted samples illuminated by a train of short saturating flashes allows the estimation of the ratio between S_0_:S_1_ and Q_B_:Q_B_^-^ in PSII (Ducruet, 2003; Zurita et al., 2005; Roncel et al., 2007). The intensity of the B band exhibited a typical four-oscillation period with maxima after the second and sixth flashes (inset of **Figure 2**). According to Inoue (1996), this pattern may suggest that in dark-adapted *P. tricornutum* cells the ratio S_0_:S_1_ and Q_B_^-^:Q_B_ is about 25:75. Thus, after one single flash, the S_1_Q_B_ centers will go to the luminescence-emitting state S_2_Q_B_^-^, generating a B band peaking at about 30–32°C (**Figure 2**, 1F), generally identified as the B_2_ band component. Two flashes induced the largest glow curve because they generate a large amount of PSII centers in both S_2_Q_B_^-^ and S_3_Q_B_^-^ luminescence states (Rutherford and Inoue, 1984). Besides, the yield from the latter recombination is higher than that from the former, by a factor of 1.7–2.0 (Rutherford et al., 1984). The much higher contribution of the second component of the B band (B_1_ band) after two flashes induces the appearance of a TL glow curve significantly shifted to lower temperatures and broadened.

To confirm that the proposed AG band observed in *P. tricornutum* cell suspensions after white light flash excitation can be identified as a typical AG band (normally induced by FR light), we have also performed TL measurements after continuous illumination of cell samples with 720 nm monochromatic light (**Figure 2**, FR/2F). After this illumination, samples were also excited with two white light flashes to ensure induction of maximal signals for B and AG bands. FR illumination generated a more prominent AG band, while the B band was reduced. FR light preferentially excites PSI and consequently oxidizes the PQ pool, thus favoring in the dark a back transfer of electrons from stromal reductants to the oxidized Q_B_ and finally to the S_2_ and S_3_ states of the manganese cluster (Ducruet et al., 2005). This overall recombination reaction leads to AG emission. The mathematical analysis of the two components found by the simulation software showed *t*_max_ values of 21 and 40°C with signal contributions of 43 and 57% for the B and AG bands, respectively (**Table 2**). These results support that the 40°C band observed in *P. tricornutum* cell suspensions after excitation with white light flashes (**Figure 2**, 2F) corresponds to the same recombination reaction which gives rise to the FR-induced AG TL band previously described (Roncel et al., 2007).

**FIGURE 3:**
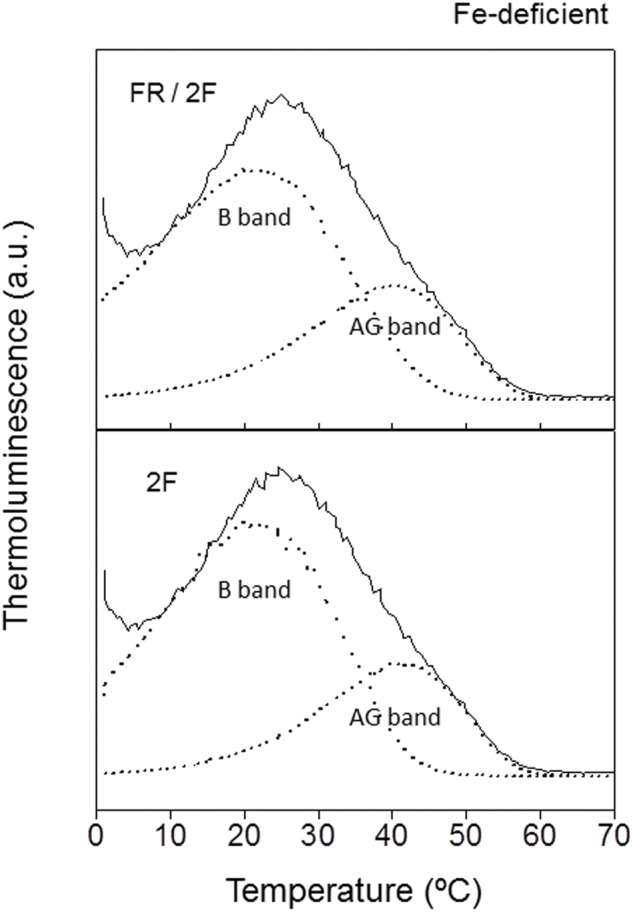
**Thermoluminescence glow curves of *P. tricornutum* cells from Fe-deficient cultures.** Typically, cell suspensions (equivalents to 15 μg Chl) from 21 days Fe-deficient cultures were incubated in the darkness for 2 min at 20°C, then cooled to 1°C for 1 min and illuminated at the end of this period with two flashes of white light (2F) separated by 1 s. Other experimental conditions as described in **Figure 2**.

Thermoluminescence was used to determine the effects of Fe deficiency on PSII electron transfer activity of *P. tricornutum* cells (**Figure 3**). Excitation of Fe-deficient *P. tricornutum* cells with two flashes at 1°C induced the appearance of a TL glow curve with significant differences in comparison with the curves obtained in Fe-replete cells: a decrease on the total TL signal intensity of about 10% and also a significant decrease of the 40°C component of the signal (AG band; **Figure 3**, 2F). The decomposition analysis of this emission curve allowed obtaining *t*_max_ values of 22 and 41°C and signal contributions of 71 and 29% for the B and AG bands, respectively (**Table 2**). Thus, although the *t*_max_ values were similar for both iron conditions, a significant increase of the signal contribution of the B band was detected (about 8%) in parallel with a similar decrease for the AG band (**Figures 2** and **3**; **Table 2**).

Thermoluminescence measurements were also performed after continuous illumination of Fe-deficient *P. tricornutum* cell samples with 720 nm (FR) monochromatic light (**Figure 3**, FR/2F). The mathematical analysis of the two components found by the simulation software showed *t*_max_ values of 20 and 39°C and signal intensity contributions of 66 and 34% for the B and AG bands, respectively (**Table 2**). Thus, the signal contributions for B and AG bands remained similar to that observed in white light excitation experiments without previous FR illumination (**Figure 3**, 2F; **Table 2**). Interestingly, FR excitation did not increase the amount of AG band in *P. tricornutum* cells cultivated in Fe deficiency, as observed for Fe-replete cells (**Figures 2** and **3**, FR/2F).

**FIGURE 4:**
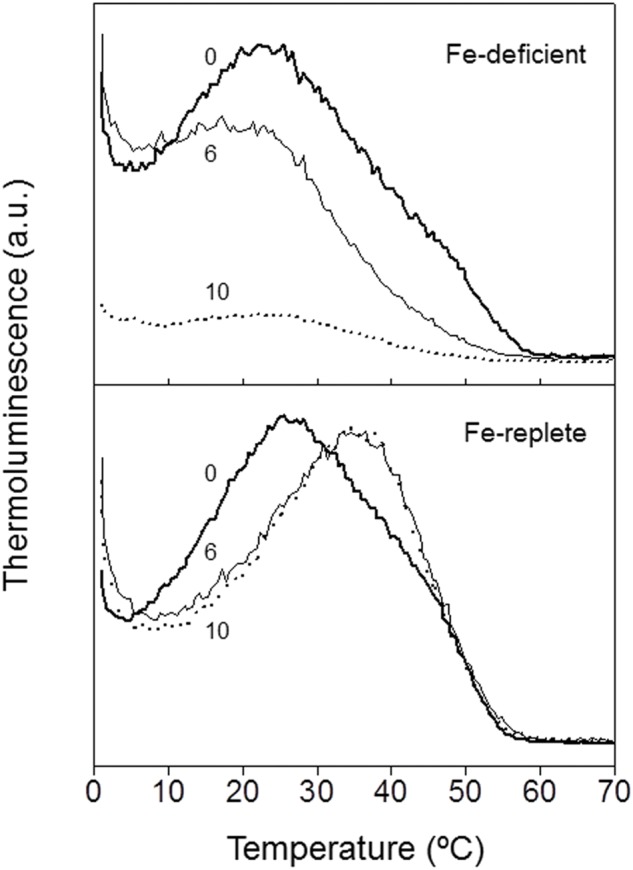
**Effect of white light illumination on TL emissions of *P. tricornutum* cells cultured under Fe-deficient and Fe-replete conditions.** Cell suspensions from 21 days cultures of both Fe conditions (Fe-replete and Fe-deficient) were harvested and suspended in the TL cuvette at the same Chl concentration. Samples were illuminated with white light of 300 μE m^-2^ s^-1^ intensity during different time periods (0, 6, or 10 min). Cell suspensions were then incubated in the darkness for 1 min at 20°C, cooled to 1°C for 1 min and illuminated at the end of this period with two flashes (separated by 1 s) of white light. Luminescence emission was then recorded while warming samples from 1 to 80°C at a heating rate of 0.5°C s^-1^. For further details see “Material and Methods” section.

The effects of high light intensity on the TL emission curves induced in *P. tricornutum* cells in both Fe-replete and Fe-deficient culture conditions have been also investigated (**Figure 4**). Cells from 21 days cultures of both Fe conditions were harvested and suspended in the TL cuvette at the same Chl concentration. Cells were then illuminated with white light of 300 μE m^-2^ s^-1^ intensity during different time periods. This medium light intensity was chosen because when using a high photoinhibitory light intensity (1000 μE m^-2^ s^-1^) not TL signal was detected for either Fe conditions (data not shown). After these illuminations, TL emission was recorded as described in “Materials and Methods” section. **Figure 4** shows the results obtained. The application of a light intensity of 300 μE m^-2^ s^-1^ during increasing time periods induced the progressive decrease of luminescence emission in Fe-deficient cells (**Figure 4**, upper). After 6 min of illumination a decrease in emission intensity of TL of about 30% was observed. However, after 10 min of light illumination, the TL signal was almost abolished. The effects of illumination in Fe-replete cells were significantly different. The application of 300 μE m^-2^ s^-1^ light during increasing time periods did not induce changes in the total intensity of the luminescence emission in Fe-replete cells (**Figure 4**, lower). However, after 6 or 10 min of illumination a significant increase of the signal contribution of the AG band was detected (from 30% to about 68%) in parallel with a similar decrease for the B band. Thus, these results suggest that Fe deficiency induced an increase in light sensitivity of PSII.

In photosynthetic cells, photoinhibitory conditions (as exposure to high light intensities) increase the probability to generate the very reactive and toxic ^1^O_2_ species (singlet oxygen) in PSII. The formation of singlet oxygen could initiate the peroxidation of unsaturated lipids in membranes (Vavilin and Ducruet, 1998). The level of lipid peroxidation in photosynthetic membranes can be measured by the HTL technique (see Materials and Methods section) (Roncel et al., 2007). Several luminescence high temperature bands (HTL bands) have been observed without prior illumination at temperatures above 60°C (Ducruet and Vavilin, 1999; Roncel et al., 2007). A broad HTL band centred near 130°C (known as the HTL2 band) is generated because of the thermal radiative decomposition of lipid peroxides that, in turn, leads to the formation of carbonyl groups in a triplet state followed by migration of excitation energy toward Chl (Vavilin and Ducruet, 1998; Ducruet and Vavilin, 1999; Vavilin et al., 2002). The amplitude of this band has been well correlated with the accumulation of malondialdehyde, an indicator of lipid peroxidation in standard chemical tests (Vavilin and Ducruet, 1998; Vavilin et al., 2002).

**FIGURE 5:**
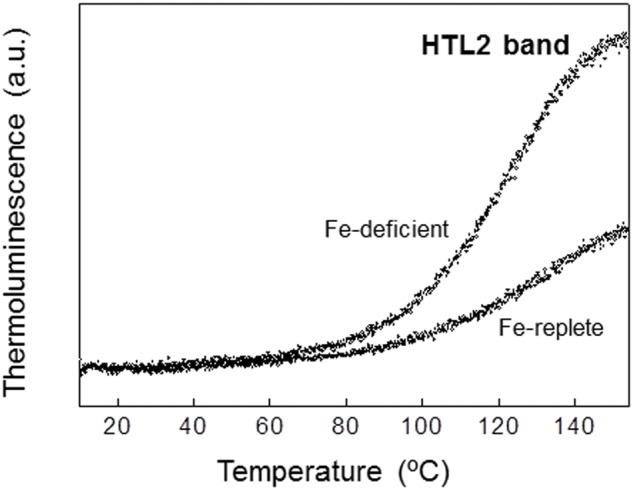
**Effect of iron deficiency on the level of peroxidation of the chloroplast lipids of *P. tricornutum.*** High temperature thermolumine-scence glow curves (HTL2 band) of *P. tricornutum* cells from both Fe-replete and Fe-deficient cultures. Cell suspensions from 21 days cultures (equivalents to 7.5 μg Chl) were adsorbed by filtration on a piece of filter paper that was pressed against the copper film, dark-incubated for 10 min at 20°C, and cooled to 10°C for 1 min. Luminescence emission was then recorded while warming samples from 10 to 160°C at a heating rate of 0.1°C s^-1^. For further details see “Material and Methods” section.

The HTL technique was applied to detect lipid peroxidation in *P. tricornutum* cells cultured in both Fe-replete and Fe-deficient conditions. The measurements have been performed using cells from 21 days growth cultures under standard light intensity conditions (20 μE m^-2^ s^-1^). **Figure 5** shows that a broad HTL2 band with maximum between 140 and 150°C was present in Fe-deficient cells (**Figure 5**). This band was significantly lower in Fe-replete cells (**Figure 5**). Thus, a very high level of lipid peroxidation was observed in cells grown in Fe-deficient conditions under a low light intensity (20 μE m^-2^ s^-1^). These results suggest that Fe deficiency may induce the appearance of acceptor-side photoinhibitory processes, and consequently, the generation of singlet oxygen at a very low light intensity, which are usually not photoinhibitory.

**FIGURE 6:**
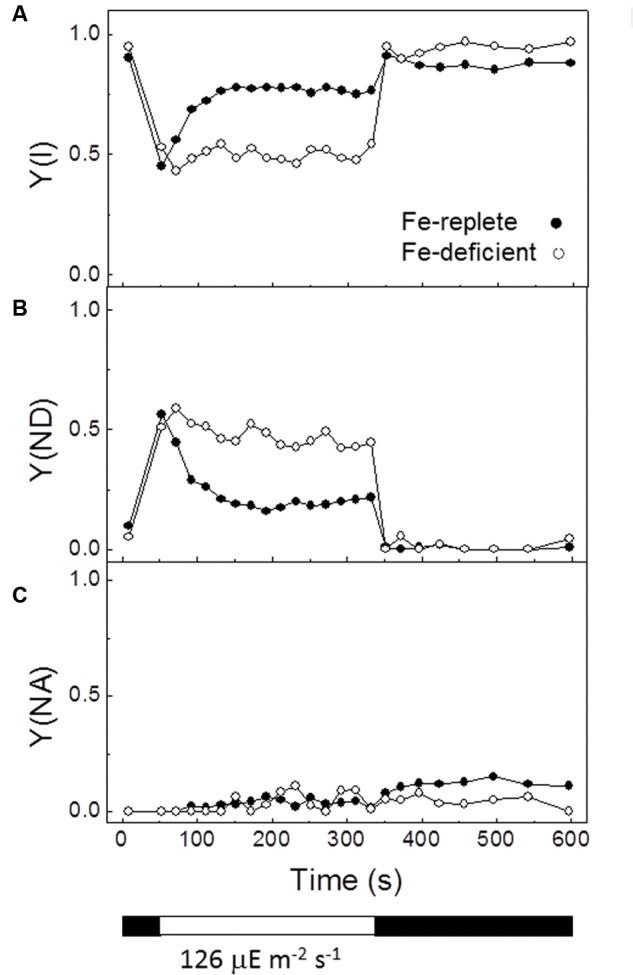
**Effect of iron deficiency on PSI activity of *P. tricornutum.*** The redox state of the PSI reaction center P700 was monitored through the changes in absorbance at 830 nm versus 875 nm and measured with a pulse-amplitude modulation fluorometer using Fe-replete and Fe-deficient cells of *P. tricornutum*. Fe-replete (*filled circles*) and Fe-deficient (*open circles*) cultures of *P. tricornutum* in the exponential phase (21 days culture) grown at 20 μE m^-2^ s^-1^ light intensity were kept in the dark for 30 min prior to the measurements. Following the initial determination of maximal oxidation of P700 the actinic light at an intensity of 126 μE m^-2^ s^-1^ was turned on and saturating pulses were applied every 20 s. After 5 min the actinic light was switched off and measurements were continued for another 5 min. **(A–C)** Changes of quantum yields of PSI, Y(I), of donor side limitations, Y(ND), and of acceptor side limitations, Y(NA), during the course of the induction curve are displayed in the figure. White and black bars below graphs indicate periods of illumination with actinic light and darkness, respectively. The curves shown in this figure are representative examples of four independent experiments.

The effect of Fe deficiency on PSI activity was also investigated by measuring the P700 redox state changes during illumination (**Figure 6**). The oxidized form of P700 displays a broad absorbance peak around 800–840 nm. Thus, it is possible to analyze its redox state monitoring changes in the absorbance at 830 nm. In dark-adapted cultures, P700 is found reduced since the acceptor side of P700, i.e., the Calvin-Benson cycle and subsequent reactions, are de-activated. Under actinic light P700 is oxidized and re-reduced by electrons coming from the PQ-pool, and thus by applying saturating pulses its ability to become oxidized and re-reduced can be determined.

Induction-recovery curves were performed in Fe-deficient and Fe-deplete cultures using red actinic light (λ 635 nm). As shown in **Figure 6A**, the calculated quantum yield of PSI photochemistry, Y(I), was substantially reduced in the Fe-deficient cultures. The loss of PSI activity proved to be caused by a lack of availability of electron donors for PSI, as shown by the higher degree of donor side limitations, Y(ND), in Fe-deficient cells (**Figure 6B**). In contrast, Fe-replete and Fe-deficient cultures were indistinguishable with respect to acceptor-side limitations, Y(NA) (**Figure 6C**). Thus, Fe limitation leads to a deficiency in PSI activity affecting specifically the supply of electrons to this photosystem in the light. However, the demand for electrons from PSI appears not to be altered.

**FIGURE 7:**
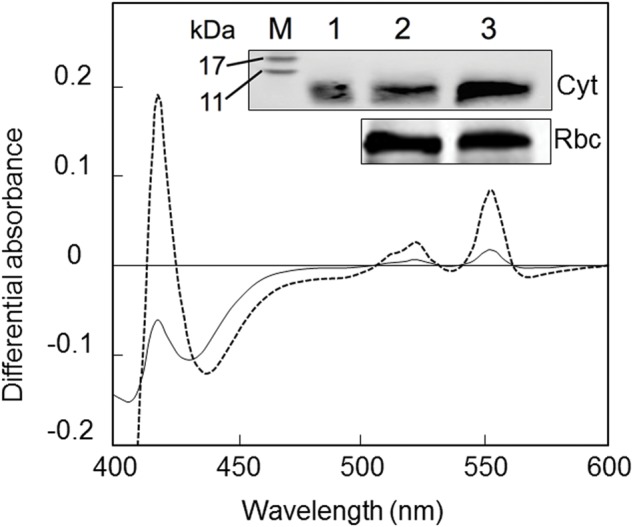
**Effect of iron deficiency on the cytochrome *c*_6_ content in *P. tricornutum* cells.** Reduced minus oxidized differential absorbance changes were recorded within the 400–600 nm spectral range in soluble cell fractions extracted of *P. tricornutum* cultures grown under Fe-replete (dashed line) or Fe-deficient (solid line) conditions. Samples were first oxidized with potassium ferricyanide to establish the baseline and then reduced by adding sodium ascorbate. *Inset*: Immunodetection of Cyt *c*_6_ in *P. tricornutum* cells cultured under Fe-replete or Fe-deficient conditions. Cell extracts of *P. tricornutum* grown under Fe-deficient or Fe-replete conditions with 20 μg of total protein were loaded into gel lanes 2 and 3, respectively. Purified Cyt *c*_6_ was loaded in lane 1 as a control. Polyclonal antibodies against Cyt *c*_6_ (Cyt) and the Rubisco large subunit (Rbc) were used. Lane M: protein molecular weight standards. The expected MW of the functional Cyt *c*_6_ is 9.7 kDa. For further details, see “Material and Methods” section.

Iron deficiency induced an important decrease in the relative content of Cyt *c*_6_ protein in *P. tricornutum* cells (**Figure 7**). Changes in the amount of Cyt *c*_6_ were determined by measuring the spectra of soluble cell fractions after 21 days of growing in Fe-replete and Fe-deficient conditions. **Figure 7** shows the ascorbate minus ferricyanide absorbance difference spectra in the region of 400–600 nm for both Fe conditions. The estimated amounts of Cyt *c*_6_ obtained from these spectra were 218 and 57 μg per grams of total cell wet weight in Fe-replete or Fe-deficient *P. tricornutum* cultures, respectively. When normalized to Chl content the values were 0.52 and 0.15 μg of Cyt *c*_6_ per mg of Chl in Fe-replete and Fe-deficient cells, respectively. Thus, under Fe-deficient conditions Cyt *c*_6_ concentration is reduced to less of 30% of the protein present in Fe-replete cells. This significant reduction of the Cyt *c*_6_ concentration as a consequence of the Fe deficiency was confirmed by Western blot analysis (**Figure 7**, inset).

## Discussion

From this work, it is shown that the culture of the marine diatom *P. tricornutum* under low Fe concentration led to a significant decline in photosynthetic and respiratory electron transfer processes, as well as to an increased sensitivity to light. To partially compensate for the negative effects of Fe limitation, it seems that a secondary electron transfer pathway, such as chlororespiration, can be activated in *P. tricornutum* cells.

Photosynthetic electron transport activity is appreciably lowered by Fe-limitation stress (Morales et al., 1991; Kudo et al., 2000; Moseley et al., 2002; Allen et al., 2008; Page et al., 2012; Urzica et al., 2012; Petrou et al., 2014; Kalaji et al., 2014a; Taddei et al., 2016). A significantly decreased photosynthetic electron transport rate (about 40% of the control) was here found in Fe-deficient cells of *P. tricornutum* (**Table 1** and **Figure 1B**), confirming previous results (Kudo et al., 2000; Allen et al., 2008). The substantial Fe requirement in both photosystems RC (three atoms for PSII; 12 atoms for PSI) and in the photosynthetic electron transport chain (six atoms for Cyt *b*_6_*f* complex, one atom for Cyt *c*_6_ and two atoms for ferredoxin molecule) (Behrenfeld and Milligan, 2012; Petrou et al., 2014) seems to be the origin of this effect. One of the objectives of this work has been to distinguish between deleterious effects of Fe deficiency on PSII and PSI activities.

Photosystem II activity of *P. tricornutum* cells was strongly affected by the culture under low Fe concentration. The inhibitory effect on the PSII photochemical activity was reflected by the decrease of the maximum quantum yield of PSII (38%; **Table 1** and **Figure 1A**) and also by the decrease on the intensity of the total TL emission signal (10%; **Figures 2** and **3**). However, the TL results obtained in this work have shown that the recombination electron transfer reaction between Q_B_ and the S_2_/S_3_ states of the manganese cluster is not affected in cells grown with low Fe concentration (**Figures 2** and **3**). The *t*_max_ for the B and AG TL bands were almost identical for the two Fe conditions (**Table 2**), thus indicating that the energetic of the electron transfer reactions involved are not affected by Fe deficiency. Thus, the low Fe concentration seems not to affect the PSII primary photochemistry in *P. tricornutum* cells.

The loss of PSII photochemical activity may be attributed to a decrease in the amount of PSII pigments, as Fe is required for their biosynthesis (Morales et al., 1991). An increased disconnection of antennae from the PSII RC in response to Fe starvation has been also proposed (Petrou et al., 2014). Due to this, the transfer of excitons to the PSII RC may be hindered, and thus, the efficiency of PSII reduction, causing a decline in the *F*_v_/*F*_m_ (**Figure 1A**). This finding is consistent with general photosynthetic responses to Fe limitation reported in diatoms (Greene et al., 1991, 1992; Geider et al., 1993; Allen et al., 2008; Lommer et al., 2012; Petrou et al., 2014), green algae (Vassiliev et al., 1995), cyanobacteria (Guikema and Sherman, 1983; Riethman and Sherman, 1988), and higher plants (Morales et al., 1991; Larbi et al., 2006; Timperio et al., 2007; Kalaji et al., 2014a). In *P. tricornutum* cells grown under Fe deficiency a significantly lower concentration of Chl *a* was observed (**Table 1**). The decreased PSII activity may be also attributed to a reduced amount of functional PSII complexes in cells (Msilini et al., 2011) or the presence of light harvesting complexes connected to inactive PSII complexes due to Fe depletion (Wydrzynski, 1982). Recently, Taddei et al. (2016) have reported that the photosynthetic capacity is severely impaired in *P. tricornutum* when Fe is limited, as demonstrated by the lower *F*_v_/*F*_m_ and rETR. These authors have proposed that the decreased maximal rETR was probably caused by a diminished capacity of carbon fixation.

Photosystem I activity has been described to be more sensitive than PSII activity to Fe-limitation (Pushnik and Miller, 1989). The results of this work have showed that the electron donation to PSI is severely inhibited by Fe deficiency. In Fe-deficient cultures of *P. tricornutum*, a significant lower quantum yield of PSI was detected (**Figure 6A**). A deficiency of donors or acceptors of PSI may be the reason for this effect. In the first case, the pool of P700, the primary donor of PSI, could not be reduced whereas in the second case the pool of P700 cannot be oxidized. The calculated quantum yield of donor-side limitations Y(ND) (**Figure 6B**) and acceptor-side limitations Y(NA) (**Figure 6C**) showed that the significant decreased quantum yield of PSI [Y(I)] obtained in Fe-deficient cultures is due to limitations on the donor side of PSI. In dark-adapted cultures, P700 is found reduced and the acceptor side of P700, i.e., the Calvin–Benson cycle and subsequent reactions, are de-activated. Under actinic light, P700 becomes oxidized and later reduced by electrons coming from the PQ pool. Under Fe-deficient conditions, P700 cannot become reduced possibly due to fewer Fe-containing electron transfer complexes downstream of the PQ pool, such as the Cyt *b*_6_*f* complex or the Cyt *c*_6_ soluble donor (**Figure 7**) (Bruce and Malkin, 1991; Greene et al., 1992; Allen et al., 2008). In particular, in *P. tricornutum* cells grown under low Fe conditions, the Cyt *c*_6_ concentration is reduced to less than 30% of the protein present in Fe-replete cells, thus presumably disfavouring the PQ pool re-oxidation. This decrease in Cyt *c*_6_ is significantly larger than the previously estimated following photochemically induced absorbance changes in whole cells (Allen et al., 2008).

An over-reduction of the PQ pool may induce the appearance of the acceptor side photoinhibition process in PSII, thus generating singlet oxygen species (Murata et al., 2007). This highly reactive form of oxygen can cause peroxidation of the membrane lipids. Analysis of HTL2 bands of TL obtained in *P. tricornutum* cells (**Figure 5**) clearly showed a much higher level of lipid peroxidation in Fe-deficient conditions, suggesting a high rate of generation of reactive singlet oxygen. Interestingly, this photoinhibitory process seems to be activated in Fe-deficient cells under a very low light intensity.

The severe decline of the photosynthetic electron transport activity induced by Fe deficiency in *P. tricornutum* cells led probably to a significant reduction of the level of synthesis of ATP and NADPH in the stroma. There is a TL band emission associated to PSII, the AG band, directly related to the assimilatory potential ([NADPH +ATP]) in the stroma (Heber et al., 1986; Mellvig and Tillberg, 1986; Palmqvist et al., 1986; Krieger et al., 1998, Roman and Ducruet, 2000). This TL AG band is observed in higher plants and algae after FR pre-illumination or, sometimes, after two or three flashes, peaking at about 45°C at a 0.5°C s^-1^ warming rate. The AG band corresponds to the fraction of PSII centers in the S_2_/_3_Q_B_ non-radiative state immediately after pre-illumination, in which the arrival of an electron transferred from stroma along cyclic/chlororespiratory pathway(s) produces the S_2/3_Q_B_^-^ radiative state that emits luminescence (Sundblad et al., 1988). The analysis of the emission curves induced by two flashes has shown the existence of an AG band of similar energetic characteristics (*t*_max_ at 40–41°C) in dark-adapted *P. tricornutum* cells grown under both Fe-replete and Fe-deficient conditions (**Figures 2** and **3**). However, it has been detected a significant decrease of the contribution of this component to the total TL intensity in Fe-deficient cells, thus suggesting the existence of a lower assimilatory potential ([NADPH +ATP]) in the stroma of these cells.

Far red light preferentially excites PSI and consequently oxidizes the PQ pool, thus favoring in the dark a back transfer of electrons from stromal reductants to the oxidized Q_B_ and, finally, to the S_2_ and S_3_ states of the manganese cluster (Ducruet et al., 2005; Roncel and Ortega, 2005). This overall recombination reaction leads also to AG emission. Interestingly, FR illumination did not increase the amount of the AG band in *P. tricornutum* cells cultured under Fe deficiency, as observed for Fe-replete cells (**Figures 2** and **3**). This phenomenon can be explained taking into account several possibilities. First, the substantial inhibition of the synthesis of Cyt *c*_6_ (this work) and functional components of the Cyt *b*_6_*f* complexes (Bruce and Malkin, 1991; Greene et al., 1992) would disfavor the PQ pool re-oxidation and consequently the appearance of PSII centers in the S_2/3_Q_B_ non-radiative state, which is initially required to generate this band. However, the oxidation process of the PQ pool was only partially inhibited under Fe-deficient conditions, as shown by polarography (**Table 1**) and fluorescence (**Figure 6**) experiments. Therefore, FR illumination should induce a detectable increase in PSII centers in the S_2_/_3_Q_B_ non-radiative state. Other possible explanation may be a dramatic decrease of the assimilatory potential ([NADPH +ATP]) in the stroma of cells grown under low Fe concentration.

The inability to generate the AG band in Fe-deficient cells illuminated with FR light could be related with the activation of cyclic/chlororespiratory electron transport pathway(s) induced by this stress condition. The back electron transfer responsible for the AG band is usually induced by warming above 35°C to activate the cyclic pathway(s). But if these pathways are already activated prior to the TL recording, the AG band emission fuses with the B band, because Q_B_ becomes reduced efficiently by stroma electrons before warming (Ducruet et al., 2011). Thus, we propose that in Fe-deficient cells of *P. tricornutum* the contribution of the 40°C AG band to the total TL emission after FR illumination not increases because it is already fused with the lower temperature B band. Fe deficiency could thus induce the activation of the chlororespiratory electron transfer pathway. This process has been proposed to be involved in protective or adaptive mechanisms of photosynthetic organisms to environmental stress conditions (Bennoun, 1982; Morehouse and Mason, 1988; Peltier and Schmidt, 1991; Rumeau et al., 2007).

The marine diatom *P. tricornutum* is highly tolerant to damage induced by exposure to high light intensities (Olaizola et al., 1994). However, the results obtained in this work have showed that this algae becomes sensitive to low and medium light intensities if is cultivated under low Fe concentration (**Figures 2** and **4**). A high level of lipid peroxidation has been detected in Fe-deficient cells under the standard culture conditions, i.e., light/dark cycles of 16/8 h and illumination with a low light intensity of 20 μE m^-2^ s^-1^ (**Figure 5**). Thus, even under these dim light conditions singlet oxygen is generated, probably due to an acceptor side photoinhibition process in PSII. Besides, the rETR observed in Fe-deficient cells at different light intensities were significantly lower than rETR from Fe-replete cells (**Figure 1**). The illumination with a light intensity of 300 μE m^-2^ s^-1^ during different time periods caused a dramatic disappearance in the TL signal amplitude in *P. tricornutum* cells grown under low Fe concentration (**Figure 4**). However, this treatment did not affect the intensity of the TL signal in Fe-replete cells, which were also capable of generating an important AG band component (**Figure 4**). These data provide strong evidence supporting the proposal that light sensitivity of the photosynthetic apparatus is substantially increased in *P. tricornutum* cells grown under low Fe concentration conditions. The partial blocking of the oxidation of the PQ pool, and the consequent induction of the acceptor side photoinhibition in PSII, might be the reason for this high light sensitivity.

Cyclic electron flow around PSII, presumably via Cyt *b*559, has been suggested earlier as a photoprotection mechanism that could retard both acceptor and donor side photoinhibition (for review, see Whitmarsh and Pakrasi, 1996). Such a cycle was shown to occur *in vivo* at high light intensities in the green alga *Chlorella pyrenoidosa* (Falkowski et al., 1986) and in the diatom *P. tricornutum*, also accompanied with the activation of chlororespiration (Lavaud et al., 2002b). Fe deficiency could induce the activation of the cyclic electron flow in PSII even at low and medium light intensities in *P. tricornutum*. However, a low synthesis of one of the proposed components of cyclic electron transfer pathway in PSII, the Cyt *b*559, would significantly decrease the efficiency of such protection mechanism.

## Conclusion

In summary, our results show that decreasing Fe concentration in the culture medium results in a significant decrease of the photochemical efficiency of both PSII and PSI complexes, as well as to an increased sensitivity to light because the activation of the acceptor side PSII photoinhibition process. We propose that the possible induction of chlorespiratory electron transfer pathway under Fe restricted conditions could partially compensate some of the metabolic negative effects of this stress condition: (1) the low levels of ATP generated by the linear photosynthetic electron transfer; and (2) the over-reduction of the PQ pool, and the consequent induction of the acceptor side photoinhibition of PSII.

## Author Contributions

MR, AL, MH, JN, and JO conceived and designed experiments; MR, MH, JN, AG-R, BN, PB-B performed experiments; MR and JO wrote the manuscript; all the authors contributed to the discussion and approved the final manuscript.

## Conflict of Interest Statement

The authors declare that the research was conducted in the absence of any commercial or financial relationships that could be construed as a potential conflict of interest.

## Supplementary Material

FIGURE S1 **Effect of iron deficiency on growth pattern of *P. tricornutum*.** Growth curves of *P. tricornutum* in Fe-replete and Fe-deficient conditions. Cells were grown in a rotatory shaker (50 rpm) at 20°C in standard ASW medium (Fe-replete culture; 12 μM Fe) and ASW medium with only 0.12 μM Fe (Fe-deficient culture). The cultures were illuminated by fluorescent white lamps at an intensity of 20 μE m^-2^ s^-1^ under a light/dark cycle of 16/8 h.
