# Supplementary figures and images for: Iron Deficiency Induces a Partial Inhibition of the Photosynthetic Electron Transport and a High Sensitivity to Light in the Diatom Phaeodactylum tricornutum
Source: Front Plant Sci. 2016 Aug 3;7:1050. doi: 10.3389/fpls.2016.01050 (PMC4971056; doi:10.3389/fpls.2016.01050)

## Slide 1
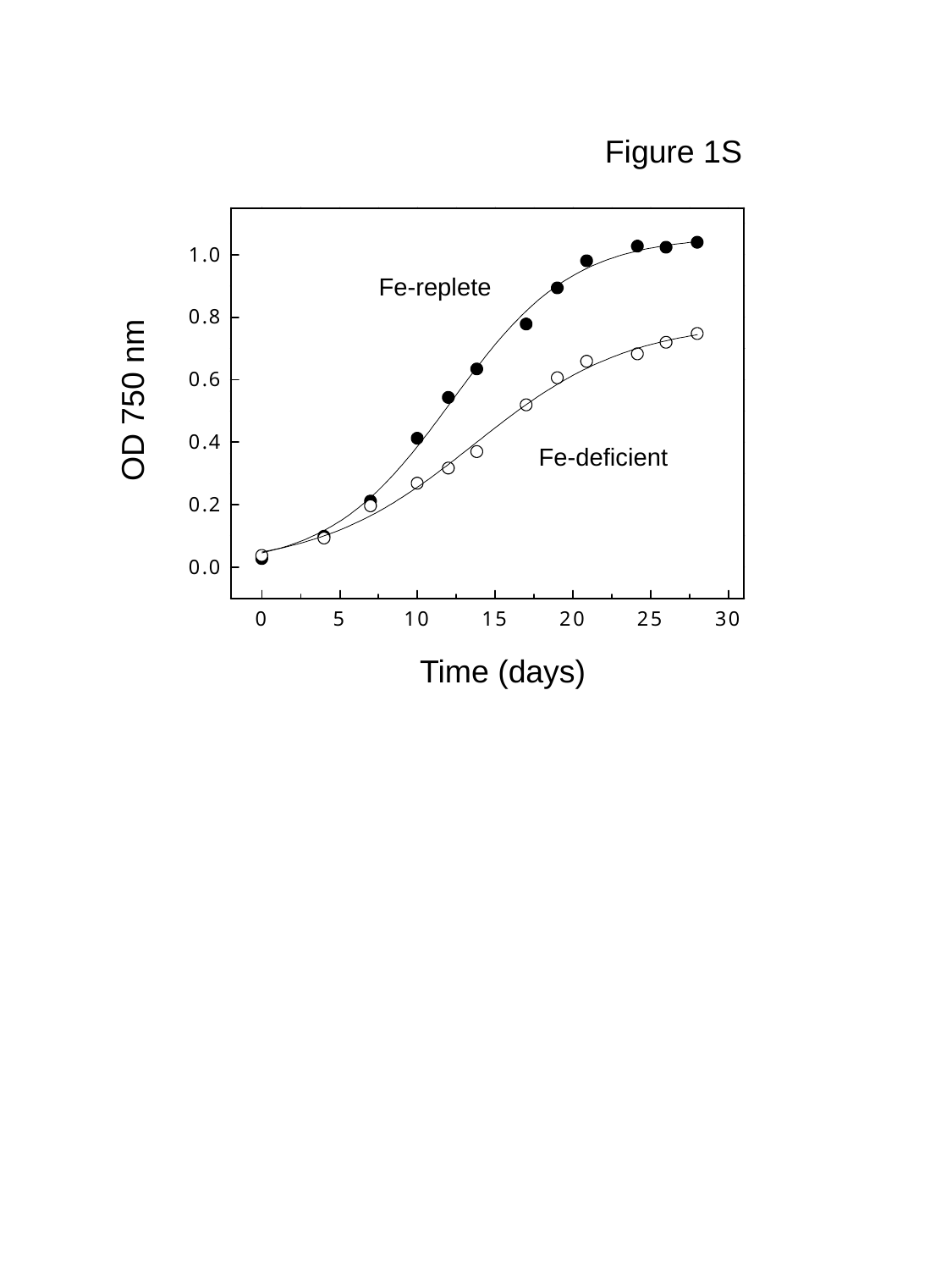

Figure 1S
Fe-replete
OD 750 nm
Fe-deficient
Time (days)

Supplement: FIGURE S1 — Effect of iron deficiency on growth pattern of P. tricornutum. Growth curves of P. tricornutum in Fe-replete and Fe-deficient conditions. Cells were grown in a rotatory shaker (50 rpm) at 20°C in standard ASW medium (Fe-replete culture; 12 μM Fe) and ASW medium with only 0.12 μM Fe (Fe-deficient culture). The cultures were illuminated by fluorescent white lamps at an intensity of 20 μE m-2 s-1 under a light/dark cycle of 16/8 h. [file Presentation_1.PPTX]
